# Comparative Evaluation of Dental Caries Score Between Teledentistry Examination and Clinical Examination: A Systematic Review and Meta-Analysis

**DOI:** 10.7759/cureus.42414

**Published:** 2023-07-25

**Authors:** Harsh Priyank, Ankita Verma, Danish Uz Zama Khan, Narendra Prakash Rai, Veena Kalburgi, Shweta Singh

**Affiliations:** 1 Department of Conservative, Endodontics, and Aesthetic Dentistry, Dental College, Rajendra Institute of Medical Sciences, Ranchi, IND; 2 Department of Pedodontics and Preventive Dentistry, Hazaribagh College of Dental Sciences and Hospital, Ranchi, IND; 3 Department Of Dentistry, Era’s Lucknow Medical College and Hospital, Lucknow, IND; 4 Department of Oral Medicine and Radiology, Faculty of Dentistry, Malaysian Allied Health Sciences Academy (MAHSA) University, Petaling Jaya, MYS; 5 Department of Periodontics and Implantology, People's College of Dental Sciences and Research Centre, People's University, Bhopal, IND; 6 Department of Public Health Dentistry, Babu Banarsi Das College of Dental Sciences, Babu Banarasi Das University, Lucknow, IND

**Keywords:** oral healthcare, dfs score, dmfs score, intraoral photographs, clinical examination, dental caries, teledentistry

## Abstract

Dental caries is a common dental health problem affecting all age groups across the globe. Accurate detection and assessment of dental caries are crucial for effective treatment and preventive measures. Teledentistry, which involves remote dental assessment using digital technologies, has shown promise as a potential tool for caries screening. This systematic review and meta-analysis aims to compare the dental caries scores obtained from clinical examinations and teledentistry assessments.

Literature searches were conducted across databases such as PubMed, Embase, the Cochrane Library, Scopus, the Web of Science, the Cumulative Index to Nursing and Allied Health Literature (CINAHL), and PsycINFO by using predefined search terms and inclusion criteria. Two reviewers separately extracted the data. The study designs, evaluation techniques, dentition types, mean scores, and follow-up times of the included studies were examined. The New Castle-Ottawa Scale was used to assess the risk of bias. Review Manager (RevMan) (computer program) Version 5.4, The Cochrane Collaboration 2020, was used for the quantitative assessment of the data. Eight studies met the inclusion criteria and were included in the review. The findings revealed that teledentistry assessments (based on intraoral photographs captured using smartphones or intraoral cameras) demonstrated comparable accuracy to traditional clinical examinations in detecting and assessing dental caries. Among the four studies that were quantitatively analysed, no significant difference was noted at p = 0.09. A mean difference of 0.64 (95% confidence interval (CI): -0.10; 1.38) suggested that clinical examination and teledentistry-based checkup were on par with each other for the detection of dental caries. The New Castle-Ottawa scale (NOS) grading indicated that the studies were of good quality. Teledentistry may be an effective approach for identifying and evaluating dental caries. However, further research is required to substantiate the findings observed in the present review.

## Introduction and background

Dental caries is a prevalent oral health condition affecting individuals of all ages worldwide. It represents a prominent global public health concern with the potential for severe complications. If left untreated, it can result in pain, infection, abscess formation, and tooth loss [1]. The prevalence of caries in permanent dentition is staggering. It affects a substantial population of approximately 2.3 billion individuals worldwide, with over 530 million children impacted [2]. Untreated dental caries and severe periodontitis were estimated to contribute to an increased incidence of systemic disease among individuals aged 60 years and older [3]. Notably, untreated dental caries in the permanent dentition emerged as the most prevalent condition, with a global prevalence of 35% across all age groups. This highlights the pervasive nature of dental caries and underscores the urgent need for effective preventive and treatment strategies [3].

Early childhood caries (ECC) represents an aggressive and severe form of caries that primarily affects children [4]. This prevalent oral health issue is a global concern [5], with an estimated worldwide prevalence rate of almost 50% [6]. The data indicate that 34.3% of children aged five to six years have already encountered dental caries [7]. It is a multifactorial disease characterised by the demineralization of tooth structures due to the interaction of bacteria, dietary factors, and host factors. Early childhood caries significantly impacts children's overall well-being, leading to various challenges such as pain, difficulties in chewing, infections, limitations in daily activities, and a decreased oral health-related quality of life [5-6]. The treatment required to address ECC can be financially burdensome, particularly when hospitalisation becomes necessary for dental interventions performed under general anaesthesia [8]. The need for an efficient preventative strategy and early intervention to lessen its impact on afflicted children and their families is underscored by the economic ramifications and resource allocation associated with managing ECC [9-10]. Effective caries management depends on early detection and prompt intervention since they can stop the disease's progression and minimise the need for invasive dental procedures [9-10].

Traditional methods of caries detection primarily rely on visual examination, tactile assessment, and radiographic imaging [7]. However, these conventional approaches have limitations, such as subjective interpretation, variation in examiner expertise, and the need for physical presence in a dental office [3]. These limitations pose challenges, especially in underserved populations, remote areas, and situations where access to dental care is limited. Teledentistry has recently emerged as a promising method for overcoming the difficulties involved with conventional dental examinations due to developments in communication technologies [11]. Teledentistry involves the remote delivery of oral healthcare services using telecommunications and digital imaging tools [12]. Teledentistry is a relatively recent advancement that leverages various forms of technology, including video conferencing, digital imaging, mobile health applications, and other remote communication tools. It allows dental professionals to interact with patients, diagnose oral health issues, provide consultations, and offer treatment recommendations, all without the need for an in-person visit. This innovative approach allows for the evaluation, diagnosis, and management of oral health conditions, including dental caries, without the need for in-person visits [12].

While the concept of teledentistry holds promise, its effectiveness and reliability in caries detection need to be thoroughly examined [13]. Several studies have investigated the accuracy and diagnostic capabilities of teledentistry compared to traditional clinical examinations [13-16]. These studies have employed various assessment methods, including intraoral photographs captured through smartphones or intraoral cameras. A better understanding of the potential and limitations of teledentistry in caries detection can be gained by analysing the collective findings from these studies. Hence, the current review was undertaken to answer the research question, "What is the difference in diagnostic accuracy between teledentistry and conventional assessment methods for the detection of dental caries?".

## Review

Methodology

Review Protocol

**Figure 1:**
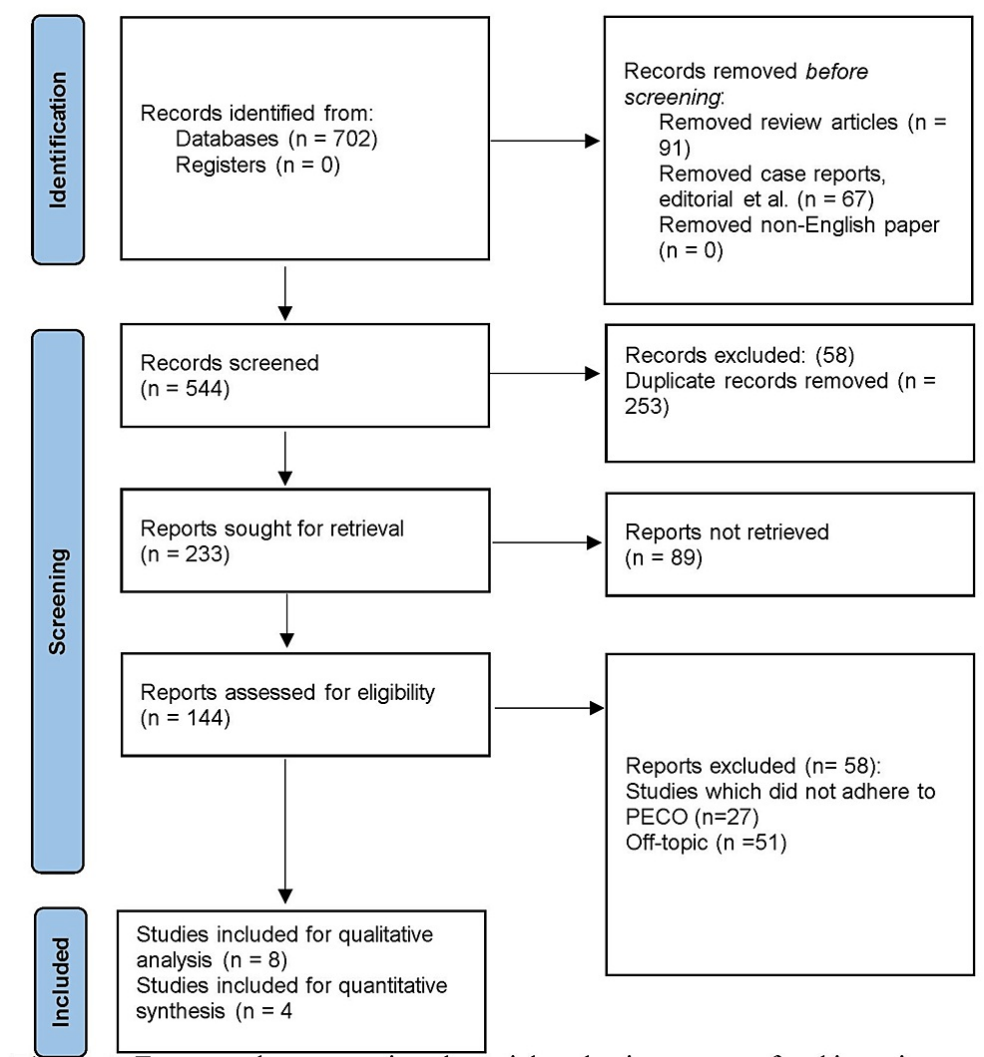
Framework representing the article selection process for the review based on PRISMA guidelines PRISMA:  Preferred Reporting Items for Systematic Reviews and Meta-Analyses; PECO: Population, Exposure, Comparison and Outcome

The present review adhered to the Preferred Reporting Items for Systematic Reviews and Meta-Analyses (PRISMA) guidelines [17] to ensure a rigorous and transparent methodology (Figure 1). This review employed a structured approach based on the Population, Intervention, Comparison, Outcomes, and Study (PICOS) framework to define the research protocol. These were Population (P): the human population of any age who underwent dental examinations for caries detection; Intervention (I): utilisation of teledentistry for dental caries detection; Comparison (C): the comparator group was the conventional or traditional clinical examination conducted by dental professionals; and Outcome (O): dental caries scores compared between teledentistry assessment and clinical examination. The review aimed to evaluate the Diagnostic Sensitivity and Specificity (DSS), mean dmft/dfs (decayed, missing, and filled teeth or decayed, filled surfaces) scores, and overall accuracy of teledentistry in identifying dental caries, as well as any differences in caries detection rates or severity between the two approaches; Study design (S): the review encompassed various study designs, including observational studies.

Studies that focused on the comparison between clinical examination and teledentistry for the detection of dental caries, conducted on human participants of any age group and with any dentition type, were included. Studies that did not involve a comparison between clinical examination and teledentistry, conference abstracts or unpublished reports, narrative reviews, opinion articles, studies focusing on non-human subjects, and those published in languages other than English were also excluded.

Database Search Strategy

The database search strategy for this systematic review, comparing dental caries scores between clinical examination and teledentistry, involved the utilisation of seven different databases (Table 1).

**Table 1:** Search protocol across different databases MEDLINE: Medical Literature Analysis and Retrieval System Online; CINAHL: Cumulative Index to Nursing and Allied Health Literature

Database	Search String
PubMed/MEDLINE	("Dental Caries"[Mesh] OR "Tooth Decay"[Mesh] OR "Caries, Dental"[Title/Abstract] OR "Dental Cavities"[Title/Abstract]) AND ("Telemedicine"[Mesh] OR "Remote Consultation"[Mesh] OR "Telehealth"[Mesh] OR "Telecare"[Mesh] OR "Teledentistry"[Title/Abstract] OR "Telemedicine"[Title/Abstract]) AND ("Clinical Examination"[Mesh] OR "Oral Diagnosis"[Mesh] OR "Dental Examination"[Mesh] OR "Oral Health"[Mesh] OR "Dental Health"[Mesh] OR "Clinical Dentistry"[Title/Abstract])
Embase	('dental caries'/exp OR 'tooth decay'/exp OR 'caries, dental' OR 'dental cavities') AND ('telemedicine'/exp OR 'remote consultation'/exp OR 'telehealth'/exp OR 'telecare'/exp OR 'teledentistry' OR 'telemedicine') AND ('clinical examination'/exp OR 'oral diagnosis'/exp OR 'dental examination'/exp OR 'oral health'/exp OR 'dental health'/exp OR 'clinical dentistry')
The Cochrane Library	((dental caries) OR (tooth decay) OR (caries, dental) OR (dental cavities)) AND ((telemedicine) OR (remote consultation) OR (telehealth) OR (telecare) OR (teledentistry) OR (telemedicine)) AND ((clinical examination) OR (oral diagnosis) OR (dental examination) OR (oral health) OR (dental health) OR (clinical dentistry))
Scopus	TITLE-ABS-KEY("dental caries" OR "tooth decay" OR "caries, dental" OR "dental cavities") AND TITLE-ABS-KEY("telemedicine" OR "remote consultation" OR "telehealth" OR "telecare" OR "teledentistry" OR "telemedicine") AND TITLE-ABS-KEY("clinical examination" OR "oral diagnosis" OR "dental examination" OR "oral health" OR "dental health" OR "clinical dentistry")
Web of Science	TS=("dental caries" OR "tooth decay" OR "caries, dental" OR "dental cavities") AND TS=("telemedicine" OR "remote consultation" OR "telehealth" OR "telecare" OR "teledentistry" OR "telemedicine") AND TS=("clinical examination" OR "oral diagnosis" OR "dental examination" OR "oral health" OR "dental health" OR "clinical dentistry")
CINAHL	(MH "Dental Caries+" OR MH "Tooth Decay+" OR TI "caries, dental" OR TI "dental cavities") AND (MH "Telemedicine+" OR MH "Remote Consultation+" OR MH "Telehealth+" OR MH "Telecare+" OR TI "teledentistry" OR TI "telemedicine") AND (MH "Clinical Examination+" OR MH "Oral Diagnosis+" OR MH "Dental Examination+" OR MH "Oral Health+" OR MH "Dental Health+" OR TI "clinical dentistry")
PsycINFO	(AB("dental caries" OR "tooth decay" OR "caries, dental" OR "dental cavities")) AND (AB("telemedicine" OR "remote consultation" OR "telehealth" OR "telecare" OR "teledentistry" OR "telemedicine")) AND (AB("clinical examination" OR "oral diagnosis" OR "dental examination" OR "oral health" OR "dental health" OR "clinical dentistry"))

The search strategy was adapted to the specific syntax and indexing terms used in each database. The keywords used were selected based on their relevance to the research topic and the PICOS framework. The Boolean operator "AND" was used to combine different concepts, while the operator "OR" was used to include synonymous or related terms within each concept. MeSH terms were employed to enhance the precision and sensitivity of the search. The keywords and Medical Subject Headings (MeSH) terms used were variations of "dental caries," "teledentistry," and "clinical examination."

Data Extraction

The data extraction strategy employed in this systematic review involved a rigorous and meticulous approach to extracting pertinent information from the selected studies. The primary objective was to gather comprehensive data on various aspects of the included studies to facilitate in-depth analysis and synthesis of the findings. The study characteristics were systematically extracted to enumerate the author(s), year of publication, study design, and region or country in which the study was conducted. Technical details such as the assessment method employed, the technological tools or platforms used, and inferences were noted.

Bias Assessment

The Newcastle-Ottawa Scale (NOS) [18] evaluated the quality and methodological rigour of the included studies. It assessed three main domains: selection of study groups, comparability of groups, and ascertainment of the outcome of interest. Each domain includes several items that are scored to determine the overall risk of bias. For each item, the study was given a specific number of stars (ranging from 0 to 9) based on the level of risk of bias. A higher number of stars indicates a lower risk of bias. The scores from the three domains were then summed to obtain the overall quality assessment of the study.

Statistical Analysis

Review Manager (RevMan) (computer program) Version 5.4, The Cochrane Collaboration 2020 was used for the quantitative assessment of the studies included. Forest plots were used to visualise the results of the meta-analysis. The random effect (RE) model was chosen based on the assumption of heterogeneity in the included studies.

Results

Study Characteristics

The initial stage of the review process yielded a total of 702 articles that were potentially relevant to the research question. Subsequently, a thorough screening process was employed to assess the eligibility of these articles based on predefined inclusion and exclusion criteria. During the screening process, duplicate articles were removed, resulting in a reduced number of unique articles. The remaining articles underwent an initial screening based on their titles and abstracts. Irrelevant articles, those not aligned with the research topic, or those clearly falling outside the scope of the review were excluded. This initial screening process further narrowed down the number of articles. Next, the selected articles underwent a detailed full-text review. The full texts were carefully assessed to determine whether they met the predetermined inclusion criteria. After this rigorous screening process, which involved multiple levels of assessment, a final selection of eight articles [19-26] was made for inclusion in the review, as seen in Figure 1.

Table 2 provides a comprehensive overview of the demographic characteristics of the sample sizes across the selected studies [19-26].

**Table 2:** Variables pertaining to the demographic characteristics of the analysed papers

Study ID	Year	Region	Sample size (n)	Age range (in years)	Gender ratio
Al Shaya et al. [19]	2022	Saudi Arabia	95	5-10	54 males
Azimi et al. [20]	2023	Australia	42	<4	19 males
Daniel et al. [21]	2017	USA	82	4-7	27 males
Estai et al. [22]	2016	Australia	100	1-65+	64 males
Golsanamloo et al. [23]	2022	Iran	20	6-12	Eight males
Kopycka et al. [24]	2013	USA	291	1-5	Unspecified
Pandey et al. [25]	2023	India	18	60-75	11 males
Purohit et al. [26]	2017	India	139	12	62 males

The studies were conducted in various regions, including Saudi Arabia, Australia, the USA, Iran, and India, reflecting a diverse representation of populations across different countries. This diversity contributes to the generalizability of the findings. The sample sizes ranged from 18 to 291. This variation in sample size allows for a broader understanding of the efficacy of teledentistry in different population settings. The age ranges of the participants varied across the studies, covering a wide range of individuals from less than four years old to 65 years and older. This broad age range indicates that teledentistry has been evaluated across different age groups, including young children, adolescents, adults, and elderly individuals. The gender ratios reported in the studies also varied, with a majority of male participants in some studies.

Table 3 provides a comprehensive summary of the eight studies [19-26] assessing the efficacy of teledentistry for caries detection.

**Table 3:** Variables pertaining to the assessed group types and the related inferences in the selected papers TG: teledentistrygroup; CG: clinical group; DSS: Diagnostic Sensitivity and Specificity; dfms: primary dentition; DMFT: permanent dentition

Study ID	Groups assessed	TG assessment method	Dentition type	Caries score (TG)	Caries score (CG)	Efficacy assessment
Al Shaya et al. [19]	TG and CG	Intraoral photographs (using a smartphone)	Primary and permanent	Primary dentition (dmfs) = 3.42±3.3; Permanent dentition (DMFT) = TG 0.69±1.1	Primary dentition (dmfs) = 3.38±3.0; Permanent dentition (DMFT) = 0.75±1.2	No significant differences were noted between the teledentistry examination and clinical assessment for dmft or DMFT.
Azimi et al. [20]	TG and CG	Intraoral photographs (using a smartphone application)	Primary and permanent	DSS= 91.2% to 100%	DSS= 44% to 88.4%	E-health screening via photographs was considered a feasible, low-cost, and sustainable option for caries detection. Specificity >95% was noted, suggesting teledental examinations to be significant during pandemic times.
Daniel et al. [21]	TG and CG	Intraoral photographs (using a smartphone)	Primary	dfs score =7.60	dfs score = 5.38	No difference (p > 0.10) was noted in caries assessment between telehealth evaluation and clinical examination when conducted by either the dentist or the dental hygienist.
Estai et al. [22]	TG and CG	Intraoral photographs (using a smartphone)	Primary and permanent	DSS = 60% to 68%	DSS = 97% to 98%	With a specificity of 60% to 68% and a specificity of 97% to 98%, smartphones were effective in dental caries screening in remote areas.
Golsanamloo et al. [23]	TG and CG	Intraoral photographs (using a smartphone)	Primary and permanent	DSS = 76.44% to 92.9%	DSS = 73.22% to 95.8%	It was observed that teledentistry could improve patient care with similar diagnostic accuracy as compared to clinical evaluations.
Kopycka et al. [24]	TG and CG	Intraoral photographs (using an intraoral camera)	Primary	dfs score = 2.19; dmfs score =1.43	dfs score =1.27; dmfs score = 0.51	When screening preschool kids for caries, examinations in the TG were equivalent to CG.
Pandey et al. [25]	TG and CG	Intraoral photographs (using a smartphone)	Permanent	DSS = 92% to 93% and 98% for both groups	DSS = 98%	When compared to a conventional clinical dental examination, teledentistry (using a smartphone) provided adequate accuracy for identifying caries in elderly individuals.
Purohit et al. [26]	TG and CG	Videographic evaluation (using a smartphone)	Primary and permanent	dmfs score = 2.46±1.91	dmfs score = 2.47±2.01	Teledentistry showed sufficient accuracy for the detection of caries in school students when compared to a conventional examination.

In terms of the assessment methods used, teledentistry predominantly relied on intraoral photographs captured with smartphones or intraoral cameras. These methods allowed for the visual examination and evaluation of dental caries remotely. The mean scores at baseline and the end of the follow-up period were reported for both the teledentistry group (TG) and the clinical group (CG). The dentition types assessed in the studies included both primary and secondary dentitions. This comprehensive approach ensured that teledentistry was evaluated for its effectiveness in detecting caries in both children and adults. Diagnostic Sensitivity and Specificity (DSS) were commonly employed to evaluate the accuracy of teledentistry.

Main Findings

Qualitative assessment of the included studies showed that teledentistry examination was as accurate as clinical examination in both primary and permanent dentistry. This suggests that teledentistry can be considered a suitable alternative to conventional diagnostic methods, especially during pandemic periods and in rural areas.

**Figure 2:**
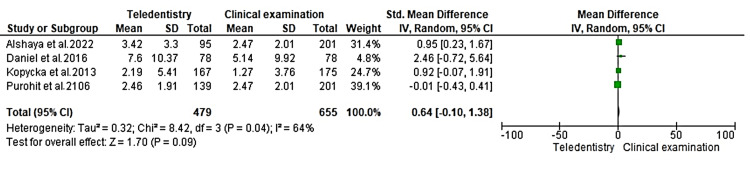
Forest plot for comparison of dental caries between teledentistry and clinical examination SD: standard definition; CI: confidence interval; IV: intervention References: [19,21,24,26]

A comparison of the dental caries assessment between telehealth assessment and clinical evaluation using Review Manager version 5.4 at a 95% confidence interval (CI) was calculated, as seen in Figure 2.

A total of 479 samples in the teledentistry examination category were compared with 655 in the traditional checkup section. Among the four studies analysed, no significant difference was noted at p = 0.09. A mean difference of 0.64 (95% CI: -0.10; 1.38) suggested that clinical examination and teledentistry-based checkup were on par with each other for the detection of dental caries. Statistical heterogeneity was assessed using the I2 test, which is 64% in the present analysis, suggesting moderate heterogeneity. This variation could be due to differences in methodological evaluation, sample sizes, and location of examination.

Risk of Bias Assessment

All studies scored 7 to 8 stars for the NOS risk of bias tool and hence were considered to be of good quality, with a moderate-to-low risk of bias.

Discussion

The significance of this review lies in the observed findings, which contribute to the growing body of knowledge on teledentistry and its potential implications for dental care. The findings demonstrate that teledentistry, utilising intraoral photographs captured through smartphones or smartphone applications, can be an accurate and effective tool for detecting caries in different populations. The studies collectively reveal that teledentistry shows comparable accuracy to traditional clinical examinations in identifying caries. This suggests that teledentistry has the potential to supplement or even replace conventional methods. The ability of teledentistry to provide adequate accuracy in identifying caries can help facilitate early detection and intervention, leading to timely treatment and improved oral health outcomes. Furthermore, the studies demonstrate that teledentistry is feasible and usable in various settings. After a brief training period, clinicians were able to effectively utilise teledentistry techniques to detect caries. This highlights the adaptability and user-friendliness of teledentistry, making it a viable option for dental professionals with varying levels of expertise, including mid-level dental professionals. By expanding the reach of dental care beyond traditional clinical settings, teledentistry has the potential to increase access to care, particularly for vulnerable populations. The findings also emphasise the importance of adequate training and standardised protocols in teledentistry implementation. The studies indicate that with proper training, clinicians can achieve high DSS, ensuring reliable and accurate assessments. This underscores the need for continuous education and quality control measures to optimise the effectiveness of teledentistry in routine dental practice.

The findings showed that the mean DMFs scores in the TG were comparable to those in the CG. This suggests that clinicians were able to accurately identify and assess dental caries from the intraoral photographs captured using smartphones or intraoral cameras. The mean dmfs scores ranged across studies, indicating variations in the severity and prevalence of dental caries in the study populations. The CG also demonstrated similar mean dmfs scores, suggesting that the teledentistry assessments were comparable to traditional clinical examinations in detecting dental caries. Similarly, the mean DFS scores in the TG were comparable to those in the CG. This indicates that the teledentistry assessments effectively identified and evaluated dental caries in both primary and secondary dentitions. The distinctions in the research populations and the extent of dental caries in those groups may be to accountable for the discrepancies in mean DFS scores between investigations. In addition to the dmfs and dfs scores, other assessment parameters were reported, such as interrater reliability (IRR) and DSS. The high inter-rater reliability scores indicated that dental reviewers consistently agreed on the interpretation of dental photographs, demonstrating the reliability of teledentistry assessments. The varying levels of sensitivity and specificity observed in the DSS results reflect the diagnostic accuracy of teledentistry in detecting dental caries, which may be influenced by factors such as the study design, the characteristics of the study population, and the methodology used in the teledentistry assessments.

Previous research has demonstrated varying levels of diagnostic accuracy between the photographic method and traditional dental examinations, with primary dentition exhibiting superior performance compared to permanent dentition [27-29]. However, the challenges posed by uncooperative behaviour exhibited by young children during dental photography may impact the overall efficacy of this method. Interestingly, one of the studies included in our analysis revealed that the use of intraoral photographs tended to be more conservative compared to visual examination [20]. The results of our systematic review corroborate the findings of a prior study, which highlighted a heightened inclination among individuals to embrace online medical protocols amidst the ongoing COVID-19 pandemic [30]. The enhanced integration of these platforms with cellular apparatus enables users to conveniently access and engage with them, regardless of time and location. Such accessibility and convenience could potentially play a significant role in fostering a positive reception and acceptance of these digital approaches for the purpose of dental screening [31].

One article [32] assessing the diagnostic performance of teledentistry compared to clinical examination found the DSS ranging from 94% to 100% and 52% to 100% for the TG. The methodology employed in their study entailed the utilisation of noninvasive dental photographs as a diagnostic tool, with a notable distinction in the assessment process between the noninvasive photography and clinical examination groups. Another research paper [33] reported DSS ranging from 60% to 68% and from 97% to 98%, respectively. Consistent with these findings, our study also demonstrated a higher diagnostic specificity compared to sensitivity. Additionally, a separate study [34] focused on comparing the performance of various smartphone models and a dedicated camera with clinical examination in detecting different grades of carious lesions in primary molars. The clinical examination phase involved direct examination of the teeth by two additional examiners who served as the standard reference. The results showed substantial agreement between noninvasive photography using a macro camera or smartphone and clinical examination. These findings highlight the potential of noninvasive dental photography methods, both using dedicated cameras and smartphones, as reliable tools for detecting and assessing dental conditions with a level of agreement comparable to clinical examination. Teledentistry bridges the gap between underserved populations and access to dental services, addressing various barriers that prevent individuals from receiving timely and appropriate oral healthcare. It eliminates geographical barriers by allowing individuals in rural or remote areas to connect with dental professionals located in urban centres. This virtual connection enables underserved populations to access dental advice, consultations, and even preventive or non-invasive treatments that would otherwise be difficult to obtain due to geographical constraints. It reaches vulnerable populations, such as the elderly, disabled, and homebound individuals, who often face difficulties in accessing traditional dental services.

Certain limitations need to be considered when interpreting the results. The sample sizes in the studies included for review were smaller, which would limit the generalizability of the findings. Additionally, the age ranges varied across the studies, making it difficult to draw conclusive conclusions for specific age groups. Further research with larger and more diverse samples is necessary to validate and extend these findings. Another limitation is the lack of standardised assessment methods and follow-up periods across the studies. The assessment methods employed to detect caries varied, including DMFS scores, DFSS scores, and DSS measures. Moreover, the specific follow-up periods were unspecified in some cases, making it challenging to compare the efficacy of teledentistry over consistent time frames. Furthermore, the studies reviewed did not provide detailed information on potential confounding variables, such as the socioeconomic status or oral health status of the participants. These factors may influence the accuracy of teledentistry in caries detection but were not accounted for in the reported findings.

## Conclusions

The results collectively indicate that teledentistry demonstrates adequate accuracy and efficacy in identifying dental caries when compared to traditional clinical examinations. The DSS measures, including dmfs scores and dfs scores, highlight the comparable precision of caries detection between teledentistry and conventional examinations. The findings of our review demonstrate that teledentistry and clinical examinations show comparable precision in detecting caries, suggesting that teledentistry can be relied upon as a reliable alternative for caries diagnosis. This finding also assures patients and dental professionals that teledentistry is not compromising the accuracy of caries detection, which is essential for proper treatment planning and preventive care. These findings suggest that teledentistry can serve as a viable alternative for caries screening and detection, potentially supplementing and improving patient care by reducing the need for in-person visits and facilitating remote evaluations. Moreover, the feasibility of teledentistry has been demonstrated through the workable techniques observed after providing brief training to clinicians. This implies that teledentistry can be successfully integrated into dental practices with appropriate clinician training and support. The potential for teledentistry to be utilised not only by highly skilled dental professionals but also by mid-level dental professionals indicates its potential for widespread implementation, particularly in areas with limited access to oral healthcare resources.
